# Common genetic variation of the *APOE* gene and personality

**DOI:** 10.1186/1471-2202-15-64

**Published:** 2014-05-20

**Authors:** Christian Montag, Lukas Kunz, Nikolai Axmacher, Rayna Sariyska, Bernd Lachmann, Martin Reuter

**Affiliations:** 1 Department of Psychology, University of Bonn, Kaiser-Karl-Ring 9, Bonn D-53111, Germany; 2 Laboratory of Neurogenetics, University of Bonn, Bonn, Germany; 3 Center for Economics and Neuroscience, University of Bonn, Bonn, Germany; 4 Department of Epileptology, University of Bonn, Bonn, Germany; 5 German Center for Neurodegenerative Diseases, Bonn, Germany

**Keywords:** Apolipoprotein, Personality, Molecular genetics, *APOE* ϵ4

## Abstract

**Background:**

A recent study yielded first evidence that personality plays an important role in explaining the influence of a prominent *APOE* polymorphism on cognitive decline and Alzheimer’s disease (AD) in elderly humans. Adding to this, two earlier studies examined this polymorphism in the context of individual differences in temperament traits in young humans with mixed results. In general, research linking the prominent *APOE* ϵ2, ϵ3 and ϵ4 variants and human personality is of special interest, because an influence of this gene and its prominent polymorphism on personality in young adulthood could be of diagnostic value to predict AD and its development in later years.

**Results:**

In the present study N = 531 participants provided buccal swabs and filled in a self-report inventory measuring the Five Factor Model of Personality. No association between common genetic variations of the *APOE* gene (in detail the genotypes ϵ3/ϵ3, ϵ2/ϵ3 and ϵ3/ϵ4) and personality could be observed. The remaining genotypes, including the high risk constellation ϵ4/ϵ4 for AD, were too seldom to be tested.

**Conclusions:**

In sum, the present study yielded no evidence for a direct link between common genetic variants of the *APOE* gene and personality in young adulthood.

## Background

Alzheimer’s disease (AD) represents one of the most challenging disorders in modern society with high costs for society and tremendous pain for both patients afflicted and their relatives [1]. Patients suffering from AD often show a dramatic decline in cognitive functioning [2] and changes in personality [3]. In a small percentage of AD patients, the disease shows a particularly early age of onset and is monogenetically inherited. In the vast majority of patients, however, AD is a multifactorial disorder being influenced by both nature and nurture [4]. In the context of molecular genetics, the most prominent genetic risk factor for this “sporadic” type of AD is a polymorphism on the apolipoprotein E (*APOE*) gene located on chromosome 19 with its ϵ4 allele being associated with higher risk for AD [5]. In addition to this, especially carriers of the rare homozygous ϵ4/ϵ4 variant show an earlier onset of AD [6]. A study by Meyer et al. [7] even reported data showing that the here discussed *APOE* polymorphism is more linked to the onset of AD compared to “whether subjects will develop AD if they survive to late old age.” (p. 322). The investigation of the human brain in the context of the *APOE* gene and AD revealed an association between the *APOE* ϵ4 allele and a higher rate of hippocampus volume loss when suffering from AD [8]. Given the prominent role of the hippocampus for memory processes (e. g. [9]) and the reported negative relation between hippocampus volume and memory performance in patients with neurological disorders (e. g. [10]), the ϵ4 allele – hippocampus volume (loss) association constitutes a biological explanation for the decline of memory functions in AD patients. Interestingly, this same study by Schuff et al. [8] observed no influence of the APOE polymorphism on hippocampus volume loss in persons not suffering from dementia or patients with mild cognitive impairment. Noteworthy, the relationship between hippocampus volume and memory ability is still a matter of debate. A meta-analysis of healthy subjects across the life-span supports the “smaller is better” hypothesis at least in children, adolescents and young adults whereas in older adults a great variability was the most striking finding [10]. Apolipoprotein E is a major target in Alzheimer disease, because it is known to modulate the amyloid-β aggregation in the brain being involved in the neurodegeneration [11]. The mentioned *APOE* ϵ4 allele is associated with a much stronger deposition of amyloid-β into senile plaques, which are often observed in Alzheimer pathology [12].

As mentioned above personality changes can be observed in patients suffering from AD [3]. AD patients can become less extraverted (socially outgoing), more neurotic (anxious, moody), less open (curious) and less conscientious. In this context a longitudinal study of interest investigated potential associations between common genetic variants of the *APOE* gene and temperament traits such as hyperactivity, motor activity, sociability and mental vitality [13]. Of note, the investigated subjects were very young and the significant associations between the common *APOE* genetic variants and the investigated traits changed across the follow-up measures. Among others the link between ϵ4 and higher motor activity was only observable in childhood, but not in young adolescence. Interestingly, the ϵ4 variant was only associated with higher sociability when investigated in adolescence (but not in childhood). A newer study [14] investigated the role of the above discussed *APOE* genetic variation and individual differences in personality measured with the Temperament and Character Inventory in a small sample of young females (N = 135). Here, no significant association turned up. This null association could be explained by the different methods in assessing individual differences in temperament traits^a^ and differences in the investigated ethnic group. Moreover, only females were included in the Tsai et al. study [14]. Noteworthy is also the small number of participants in this study [14]. It is well known that personality is influenced by a large number of genetic variants with small impact. As a consequence larger sample sizes are needed to carve out potential main effects of genetic variants [15].

Dar Nimrod et al. [16] extended the findings from these first pioneer studies by reporting that elderly humans carrying at least one *APOE* ϵ4 allele suffered more often from cognitive decline and AD, when being high neurotic or high extraverted. Dar-Nimrod et al. [16] explained the results as follows: Neurotic or high anxious persons tend to have lower hippocampus volumes (see also a review [17]), which could be a result of an elevated activity of the hypothalamic-pituitary-adrenal-axis resulting in glucocorticoid induced brain atrophy. Therefore, lower hippocampus volumes in healthy participants together with a genetic risk for AD could lead to an earlier decline of cognitive functions and higher AD incidence. The Extraversion result was not hypothesized by the authors, but could be explained by the idea that extraverted humans exercise their cognitive functions via social interactions. The older a person gets, the smaller the chances for social interactions are, because large parts of one’s own social network are naturally getting smaller.

When summarizing the findings of the literature, it becomes clear that the *APOE* gene might play a role in personality, although this influence might be age dependent. The effects of common genetic variation of the *APOE* gene might change strongly across childhood to adulthood, because personality does not stabilize until the age of 30 [18]. It is also conceivable that stronger effects of *APOE* would be observed in adulthood, as heritability estimates for a range of traits increase from childhood to adulthood [19].

Given the large interest in finding early markers for AD, we investigated in the present study if the common genotypes of ϵ3/ϵ3, ϵ2/ϵ3 and ϵ3/ϵ4 of the *APOE* gene explain individual differences in personality. Given the small literature in the field, we investigated a much larger and mixed sex sample of young adults compared to the study by Tsai et al. [14] utilizing the same personality questionnaire as in the study by Dar-Nimrod et al. [16].

## Methods

### Participants

N = 531 healthy Caucasian participants (n = 172 males and n = 358 females, one gender information is missing; age = 21.57, SD = 3.50, range 17–52) were recruited in different lectures of the University of Bonn. All participants filled in a self-report measure of the Five Factor Model of Personality (NEO-FFI, [20]) in the German translation [21]. Moreover all participants provided buccal swabs for genotyping the *APOE* polymorphism. Written informed consent was given by all participants. Moreover, the study was approved by the local medical ethic committee, University of Bonn.

### Self-report measure

The questionnaire NEO-FFI [20] measures the so called Big Five of Personality. These dimensions originally have been derived by factor analysis, using a lexical approach. The dimensions are called Openness to Experience, Conscientiousness, Agreeableness, Extraversion and Neuroticism. Each dimension is measured with twelve items being scored on by a five point Likert scale ranging from “strongly disagree” to “strongly agree”. In the present study we computed scale means for each dimension (with a range from 1 to 5). Of special interest in the AD literature are the personality dimensions Extraversion and Neuroticism [16]. Extraverted humans tend to be outgoing, active, assertive and seek social interactions. Neurotics are anxious, they tend to have feelings of guilt and are emotionally unstable.

### Genotyping

Automated purification of genomic DNA was conducted by means of the MagNA Pure^®^ LC system using a commercial extraction kit (MagNA Pure LC DNA isolation kit; Roche Diagnostics, Mannheim, Germany). Analysis of the *APOE* polymorphism(s) was conducted with real time polymerase chain reaction (PCR) on a Light Cycler System by Roche. Primers and hybridization probes were provided by TIBMOLBIOL, Berlin.

### Statistical analyses

After genotyping the whole sample, we excluded the extremely rare genotype groups – namely ϵ2/ϵ2, ϵ4/ϵ4 and ϵ2/ϵ4 – from the statistical analyses. As one can see in the result section, the occurrence of these genotypes is such rare that statistical analyses are not meaningful with the current sample size. With the remaining sample (n = 510 out of N = 531) the personality analyses were conducted. Here, we computed a MANOVA with the five personality dimensions as dependent variables and the remaining three genotypes of interest as the independent variable (ϵ3/ϵ3, ϵ2/ϵ3 and ϵ3/ϵ4). Given some associations of age and gender with personality we controlled for these variables in additional analyses (computed with a MANCOVA). As age and gender did not turn out to confound the gene-personality associations, we only report the results of the MANOVA in the following.

## Results

### Genotyping

Genotyping resulted in the following distribution, which fits with the distribution of the *APOE* polymorphism in the literature: the most common genotypes, which were used for the statistical analyses are ϵ3/ϵ3 (n = 351), ϵ2/ϵ3 (n = 63) and ϵ3/ϵ4 (n = 96). The genotypes ϵ2/ϵ2 (n = 6), ϵ4/ϵ4 (n = 8) and ϵ2/ϵ4 (n = 7) were rare and were excluded from the ensuing analyses. The genotype frequencies were in Hardy-Weinberg Equilibrium (χ2 = 3.537, *df* = 3, *p >* 0*.*05).

### Personality dimensions, age and gender

Females reported to be significantly more neurotic (F_(1,507)_ = 23.42, p < .001), more agreeable (F_(1,507)_ = 31.70, p < .001) and more conscientious (F_(1,507)_ = 5.31, p < .05). A significant negative correlation could be observed between age and Extraversion (r = -.13, p = < .01^b^) and a significant positive association between age and Openness (r = .17, p < .001). No other significant association was observed in this section.

In Table 1 means of the personality dimensions for the total group and split up for males and females are reported. Moreover, we include information on the reliabilities (internal consistencies assessed by Cronbach’s Alpha) of the personality dimensions in an additional column.

**Table 1:** Means and standard deviations of the personality dimensions and internal consistencies

	**Total sample**	**Male sample**	**Female sample**	**Internal consistencies**
Neuroticism	M = 2.66 (SD = 0.62)	M = 2.47 (SD = 0.62)	M = 2.75 (SD = 0.60)	α =. 85
Extraversion	M = 3.49 (SD = 0.48)	M = 3.45 (SD = 0.47)	M = 3.51 (SD = 0.49)	α = .76
Openness to Experience	M = 3.57 (SD = 0.53)	M = 3.55 (SD = 0.58)	M = 3.57 (SD = 0.50)	α = .75
Agreeableness	M = 3.71 (SD = 0.47)	M = 3.54 (SD = 0.48)	M = 3.78 (SD = 0.44)	α = .75
Conscientiousness	M = 3.71 (SD = 0.57)	M = 3.63 (SD = 0.60)	M = 3.75 (SD = 0.55)	α = .85

### *Common genetic variants of* APOE *and personality*

As the results controlling for the variables gender and age (separately or together) did neither reveal gene by gender interaction effects on personality nor change the results significantly by considering age as a covariate, we report here the MANOVA, investigating the main effect of the common genetic variations of *APOE* on personality without considering gender as a second independent variable or age as a covariate. In short: No significant influence of the *APOE* polymorphism could be observed on any of the personality dimensions. The results of the MANOVA are presented in Table 2. Of note, even the inclusion of all genotypes (including the three rare genotype groups) as independent variables did not yield any significant influence of *APOE* on personality. For future research endeavors, we present the means and standard deviations of each personality dimension depending on the genotypes (ϵ3/ϵ3, ϵ2/ϵ3, and ϵ3/ϵ4) under investigation in Table 3.

**Table 2:** **The influence of common genetic variations of the *APOE *** **gene on personality (results of MANOVA analysis)**

Neuroticism	F_(2,507)_ = 1.22, p = .30
Extraversion	F_(2,507)_ = 2.16, p = .12
Openness to Experience	F_(2,507)_ = 1.17, p = .31
Agreeableness	F_(2,507)_ = 0.55, p = .58
Conscientiousness	F_(2,507)_ = 0.74, p = .48

**Table 3:** **Means and standard deviations of the personality dimensions depending on the *APOE *** **genotype**

**Personality dimension**	**ϵ3/ϵ3 (n = 351)**	**ϵ2/ϵ3 (n = 63)**	**ϵ3/ϵ4 (n = 96)**
Neuroticism	2.65 (0.63)	2.76 (0.64)	2.62 (0.54)
Extraversion	3.50 (0.49)	3.39 (0.45)	3.54 (0.48)
Openness to Experience	3.59 (0.53)	3.57 (0.57)	3.49 (0.50)
Agreeableness	3.70 (0.48)	3.76 (0.46)	3.72 (0.42)
Conscientiousness	3.72 (0.57)	3.76 (0.63)	3.65 (0.54)

## Discussion

The present study investigated a potential influence of common genetic variation of the *APOE* gene on human personality. This is of importance, because the ϵ4 allele of this gene is associated with increased risk for developing AD, and personality has been suggested to moderate the association between the ϵ4 allele and AD [16]. Adding to this, two further studies searched for main effects of common genetic variation of the *APOE* gene on personality with mixed results [13,14].

However, no significant effect of the investigated *APOE* polymorphism on personality could be detected in the present research, therefore making it unlikely that this polymorphism plays an important role for human personality in young adulthood. If detected, a positive association between *APOE* variants and personality would have been highly interesting from a diagnostic point of view to predict AD. Of interest, a personal communication via email between Ilan Dar-Nimrod and the principal investigator of this study yielded the additional information that no main effect of the *APOE* polymorphism could be observed on the personality dimensions in their sample consisting of N = 602 elderly humans [16]. This underlines the null findings of the present research and the findings by Tsai et al. [14].

Despite this we cannot rule out that polymorphisms on the *APOE* gene, together with other genetic variations or certain environmental influences, may shape human personality. Studies investigating such gene by environment effects or epigenetics in the context of personality will be of highest interest. Of note, the *APOE* ϵ4 allele is also associated with cardiovascular disease, whereas “…higher intake of fat in the Western diet may be partly responsible for the increased risk of AD associated with *APOE* ϵ4…” [22]; p. 729. As individual differences in both exercise behavior [23] and dietary behavior [24] have been also associated with personality, these variables might constitute interesting further factors interacting with *APOE* ϵ4 in the context of the present research topic.

Some more limitations of the present study need to be mentioned. First, we applied just one (although very prominent) measure of human personality stemming from a lexical approach [20]. More biologically oriented personality inventories such as the *Affective Personality Neurosciences Scales (ANPS)* could have yielded other results [25]. Second, our results are preliminary, because the highest risk constellation for AD – namely the homozygous ϵ4/ϵ4 genotype - occurs very rarely and could not be properly investigated in the current sample. Clearly huge sample sizes are needed to obtain a sufficient number of ϵ4/ϵ4 carriers to reveal an influence of this special genotype on human personality. Future studies should investigate the interplay of *APOE* polymorphisms, personality, hippocampus volume and memory functions in a longitudinal design using multiple age cohorts.

## Conclusions

In sum, the present study provides no evidence for a role of the prominent *APOE* polymorphism on human personality in young adulthood.

## Endnotes

^a^Keltikangas-Järvinen et al. included mothers’ assessments of their children and in parts self-report data

^b^The values of the personality scales deviated from a normal distribution (tested with a Kolmogorow Smirnow test, Shapiro Wilk tests revealed the same results with the exception for Openness). Spearman’s Rho would have provided a non-significant result, here (Spearman’s rho = -.04, p = .38). Of importance for the following analyses in the result section: results did not differ when non-parametric tests were conducted. Therefore, and after visual inspection of the data (resembling normal distributed data), we decided to provide only data with parametric testing.

## Competing interests

The authors declare that they have no competing interests.

## Authors’ contributions

CM, NA and MR designed and planned the study. MR and CM carried out the molecular genetic analyses. LK, RS, BL, NA and CM collected the personality and genetic data in the field. These authors also coordinated the study. Moreover, LK, RS, and BL did the data processing. CM carried out the statistical analyses. CM, LK, RS, BL, NA and MR drafted the manuscript. All authors read and approved the final manuscript.
